# The Significance of Pains in the Pelvis and Abdomen

**Published:** 1907-06

**Authors:** W. A. Crowe

**Affiliations:** Atlanta, Ga.


Fulton County Medical Society, April 25, Dr. Claude E. Smith in the chair:

### THE SIGNIFICANCE OF PAIN IN THE PELVIS AND ABDOMIN.

Dr. W. A. Crowe.

As a diagnostic feature great stress is placed on these pains. Age is an important factor to be considered before reaching any conclusion. In a child abdominal pain suggests the possibility of lung involvement. In a person over 40 years of age and persistent, we think of a malignant growth. In the upper abdomin pain may be caused by involvement of gall bladder, liver, stomach, duodenum, etc. Inflammation of the liver or its congestion may cause pain. Along with gall gladder pain we have other symptoms which have to be considered carefully in making a diagnosis. It may simulate appendicitis. Familiarity with the origin and course of the nerve supply of these organs is very important as the pains are frequently referred. Gastro-intestinal trouble may be caused by an impacted gall stone. Pain in the liver is usually referred to the back or right shoulder or is located immediately over the liver. They are nearly always limited to the upper abdomin.

The pain of appendicitis is at first diffused in upper part of abdomin, later becomes localized at McBurney's point. It should always be borne in mind that the appendix may be misplaced, and that the severity of the pain does not always indicate the gravity of the attack. Pains simulating chronic appendicitis may sometimes be relieved by a properly fitting suspensory bandage. The differential diagnosis between appendicitis and acute salpingitis is often difficult to make. In the former the pain extends up rather than down, while in tubal trouble it is more likely to be confined to the pelvis. The history is usually not sufficient without a digatal examination and even then we may be in doubt. Here again age is important. Malignant disease is to be expected in the old, but in the young one of the common causes of pain in the lower abdomin in cystic degeneration of the ovary. Next in frequency is involvement of the tubes and prolonged ovary and perhaps pelvic peritonitis with adhesions. The operation many times is necessary to prove the diagnosis.

Interstitial tumors within the walls ofthe uterus may cause pain and much nervous distress. Ruptured ectopic pregnancy is attended with pain like that of acute fulminating peritonitis.

Discussion—Dr. Claude Smith related history of a hospital patient with severe abdominal pain which was diagnosed fulminating appendicitis. At autopsy the viscera was matted together with a general purulent exudate, but the appendix was not the cause of the trouble. After a careful search a small perforation was found in the duodenum.

Dr. E. C. Cartledge mentioned the danger of mistaking neuralgia of the abdominal wall for appendicitis and gave the histories of several patients illustrating different kinds of abdominal pain.

Dr. L. C. Rouglin said he was glad to hear Dr. Crowe classify the abdominal pains, for, as a rule, the medical profession does not give sufficient study to this subject but too readily jumps at conclusions. It should be remembered that the absence of pain in the abdomin may sometimes be a dangerous symptom, and that localized pain may not be a true guide as to the seat of the pathological condition.

Dr. W. B. Armstrong mentioned the fact that pain limited to the lower right side of the abdomin might be found in the beginning of typhoid fever and be easily mistaken for appendicitis.

Dr. M. A. Purse reported the history of a child threatened with "spasms," as the mother expressed it, on account of excruciating abdominal pain caused by a mass of undigested orange pulp and a small gold ring which were removed from the bowel by an enema. Suspensory bandages had also relieved two of his patients of abdominal pain within the last month.

Dr. I. C. Smullyan had recently had a patient with most severe pain due to an open safety pin in the lower part of the rectum.

Dr. C. R. Andrews mentioned membraneous colitis which is known to be a frequent cause of pain sometimes mistaken for appendicitis and urged that a careful search be made for casts in doubtful cases.

Dr. Crowe (closing) said Pott's disease should be remembered as a cause of abdominal pain.

